# Wetland characteristics affect abundance and diversity of wintering birds: A case study in South‐Western Iran

**DOI:** 10.1002/ece3.9558

**Published:** 2022-11-21

**Authors:** Mansoureh Malekian, Roghayeh Salarpour, Mehrdad Ranaie

**Affiliations:** ^1^ Department of Natural Resources Isfahan University of Technology Isfahan Iran

**Keywords:** drought, normalized difference water index, population trends, standardized precipitation index, Waterbirds

## Abstract

Water availability is an important driver of bird population change, and its effects are likely to increase in coming decades under climate change. Here we assess effects of temperature, precipitation, and water area on wintering bird populations in Miyangaran Wetland in southwestern Iran. Modeling methods including, generalized linear model (GLM) and hierarchical partitioning were used to examine the relative importance of variables. The number of wintering species, inhabiting the wetland, varied among years, ranging from 10 to 48 species. The total number of wintering birds showed a significant decreasing trend. A significant increasing trend was obtained for shorebirds, while waterfowl species were significantly decreased. The GLM showed that species abundance, richness, and diversity were significantly correlated with the standardized precipitation index (SPI), annual precipitation, and normalized difference water index (NDWI). Hierarchical partitioning analysis also identified NDWI, SPI, and annual precipitation as the most important variables with average independent effects of 35, 36 (*p* < .01) and 17% (*p* < .05), respectively. Our results revealed that the water area plays a major role in determining the structure of bird diversity and abundance, affecting both waterfowl and shorebirds.

## Graphical abstract

Results showed that the number of waterfowls and shorebirds were significantly decreased in the period of 2000 to 2019. Water availability is an important driver of bird population change and its effects is likely to increase in coming decades under climate change.

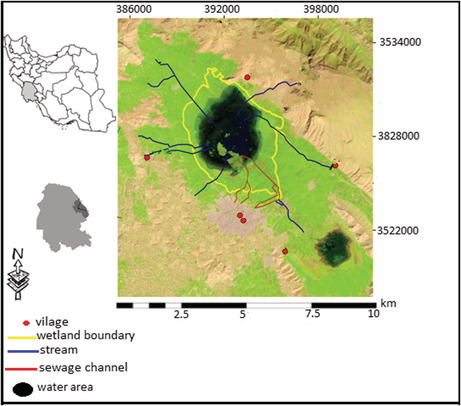

## 1 INTRODUCTION

*Wetlands are important* features in the *landscape* that provide numerous beneficial services for humans and wildlife (Hu et al., 2017). Wetlands play a crucial role in maintaining bird communities by supplying abundant resources. Unfortunately, global wetlands continue to disappear (Davidson, 2014), and available habitats for bird species are changing due to human‐induced climate change (Haig et al., 2019).

Climate change may be defined as a change in weather pattern over time that occurs due to natural changes and human activities. Several issues such as increased heat, declining water supplies, and drought are linked to climate change (Bhaga et al., 2020; Campbell et al., 2009). However, humans directly change the dynamics of the water cycle through dams constructed for water storage and through water withdrawal for industrial, agricultural, or domestic purposes (Dai, 2011; Wanders & Wada, 2015). Water stress caused or intensified by human activities, including increased demand, outdated water management, climate change from anthropogenic greenhouse gas emissions, growing energy and food production, intensive irrigation, diminished supplies, and land use change has been called *anthropogenic drought* (AghaKouchak et al., 2015). This is a multidimensional and multiscale phenomenon governed by the combination of natural water variability, climate change, human decisions and activities, and altered microclimate conditions due to changes in land and water management (IPCC, 2022).

The environmental impacts of *anthropogenic* drought are seen around the world, affecting ecosystem structure and function (Campbell et al., 2009). For example, changing the precipitation regime and reduced surface water area have important implications for avifauna, including wintering birds (e.g. Herrando et al., 2019; Spooner et al., 2018). Wintering species simply require open water as habitat and many need free water for intake. Further, for many birds, precipitation is a major driver of vegetation productivity, i.e., flower, seed, and fruit production and insect abundance (Liu et al., 2020), which are important food resources (Selwood et al., 2018) for birds. However, avifauna with different life histories and behavioral characteristics may vary in response to habitat characteristics (González et al., 2009; Malekian et al., 2021). Birds inhabiting wetlands are especially sensitive to habitat conditions and might be expected to adversely respond to changing wetland characteristics (Haig et al., 2019). For example, waterfowls and shorebirds are varied in selecting an optimum habitat (Maclean et al., 2007), and wetland characteristics such as wetland size, water depth, water level fluctuation, salinity, and vegetation cover affect the use of wetlands by waterfowls and shorebirds (Ma et al., 2010; Tavernia et al., 2021). Several studies have determined the key factors, affecting the diversity of wetland bird communities. It has been reported that fluctuations in the water area affect the distribution and abundance of shorebirds and waterfowls (Tavernia et al., 2021).

The abundance of waterbirds was positively correlated with the size of the water body (González et al., 2009), and it has been shown that waterfowl's abundance and species richness depend on the size, shape, and depth of water bodies (e.g. Baschuk et al., 2012; Rajpar & Zakaria, 2011). Significant reduction in the wetland size due to land use change, human construction, and built‐up areas has reduced the natural habitats for birds, as an example, the diversity of waterbirds dropped with higher roads and building surface coverage around a wetland (Xu et al., 2022). Determining the population fluctuation of wintering birds in wetland habitats and the effects of wetland characteristics on bird assemblages are important to regulate human activities and minimize the negative impacts. Such information is lacking in many parts of the world, including Iran.

Iran is located on the Central Asian flyway (CAF), which comprises several important migration routes of wintering birds, most of which extend from the northernmost breeding grounds in Siberia to the southernmost non‐breeding wintering grounds in West Asia and India (CMS, 2014). Here we evaluate the diversity and abundance of wintering birds in Miyangaran wetland, one of the major bird habitats in Khuzestan province, southwestern Iran. In this study, our principal objective is to determine the relationship between wintering bird assemblages and the wetland characteristics and whether variation in temperature, precipitation, and water area affect the wintering assemblages (waterfowls vs. shorebirds). We predicted that the wetland characteristics that have changed over the last decades, due to land use change and human‐induced climate change, have affected bird diversity and abundance of the wetland. We hypothesized that waterfowls and shorebirds respond differently to the changing wetland characteristics.

## 2 MATERIALS AND METHODS

### 2.1 Study area

**FIGURE 1:**
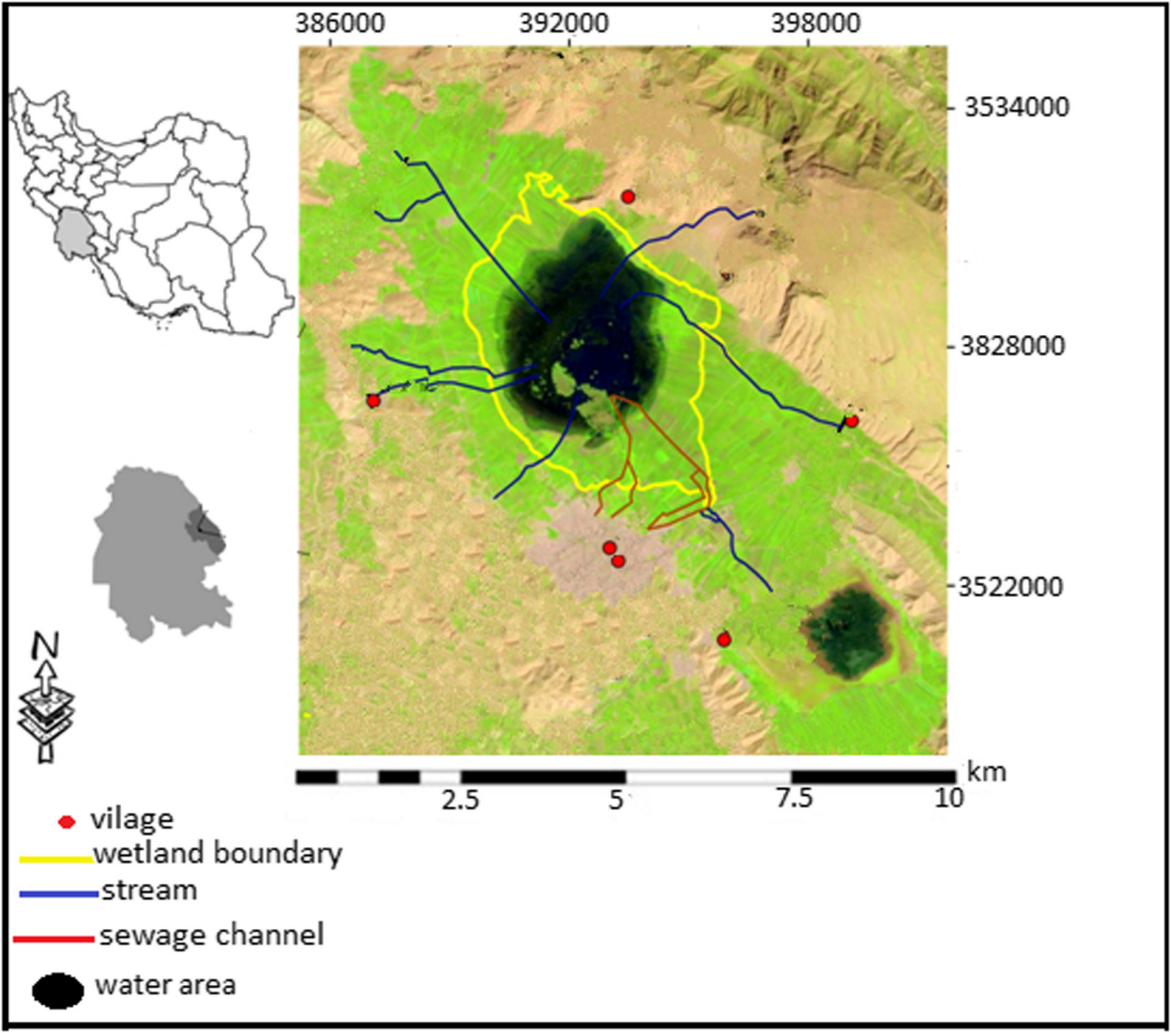
The geographic location of Miyangaran wetland in south western Iran.

Miyangaran wetland with an area of about 2240 hectares is located in the Izeh plain in the northeast of Khuzestan province, southwestern Iran (Figure 1). The elevation ranges from 822 to 845 m above the sea level, and the wetland depth is about 5 m. The study area has a subhumid temperate climatic condition with cold winter and moderate summer, thus experiencing a wide range of temperatures from a maximum of 45°C in summer to −4°C in winter. The mean relative humidity varies from a minimum of 18% in June to a maximum of 84% in December. According to Izeh meteorological station, the average rainfall in the region is 637 mm and the average annual temperature is 23°C and most rainfall occurs in autumn and winter (Esmaeili et al., 2019).

Miyangaran is an inland wetland based on the Ramsar Convention of International Wetlands. It is an important wintering habitat for migratory birds in the Middle East and recognized as an IBA site (Behroozi‐Rad, 2017). Thousands of birds visit the wetland annually and stay during the winter. The wetland, however, is one of the major water resources in Khuzestan province. Water utilization for agriculture has a long history in the region, and the progressively increasing population, in the last decade, has accelerated the water demand, resulting in consecutive drying of wetland (Kalantari et al., 2009). Other issues such as the conversion of land into agriculture and sewage and effluents from urban and agricultural areas are threatening the life of the wetland (Hosseini et al., 2014).

### 2.2 Bird census and diversity indices

In this study, wintering bird population trends were estimated, using 20 years (2000–2019) monitoring data from the Department of Environment, Khuzestan province. The annual census for migratory birds in Iran begins in mid‐winter (December 20–January 20), based on the International Waterbird Census (IWC). It is a site‐based counting scheme for monitoring waterbird numbers, organized since 1967 by Wetlands International (2010). In order to ensure consistency of bird monitoring methodology and similar coverage from year to year, all counts at the site were performed in the same way every year. The boundary of the counted area, the route followed by the counter, and the vantage points were marked on the map. The bird census area includes the area delineated by the yellow wetland boundary in Figure 1.

Before the count started, a preliminary scan was made with binoculars, and the overall number of birds and the proportion of each species were rapidly assessed. A Swarovski 10 × 40 binocular and an 80 mm (60×) spotting scope (Swarovski Optik) were used for bird identification and counting. Bird counts were conducted in clear, sunny weather from 6 am to 6 pm. All bird counts were conducted by the same field observers to minimize observer bias. Flocks were scanned several times, and birds counted one or two species at a time. If time allowed, repeated scans were used to obtain a consistent estimate, i.e., to improve the precision of the count. Different bird species were identified, and the number of individuals representing each bird species was recorded. Bird species were assigned into two different groups of waterfowl and shorebirds, based on field observations and the literature (e.g. Mansoori, 2010).

Indices of species richness, species diversity, and evenness were used to explore the complexity of the avifaunal community, inhabiting the wetland in different years. The Margalef's index of species richness, the Shannon‐Wiener diversity index, and the Camargo's evenness index were calculated, using Ecological Methodology v. 7.2. (Kenny & Krebs, 2001). Each one of these indices may provide meaningful understanding of the wetland's biodiversity changes. However, the results of these indices are not completely the same. For example, the species richness is biased toward rare species, while the species evenness is biased toward dominant species (Roswell et al., 2021). Species richness alone cannot be utilized to estimate the species diversity of an area or there are dissimilarities between the trends of evenness and biodiversity indices (Goudarzian & Erfanifard, 2017). Therefore, it is recommended to apply a combination of richness, evenness, and biodiversity to investigate the species diversity of waterbirds (Duelli & Obrist, 2003). In Iran, for example, the Margalef's index of species richness, the Camargo index of evenness and the Shannon‐Wiener index of biodiversity are suggested for aquatic birds (Goudarzian & Erfanifard, 2017).

### 2.3 Climate data

To understand the climate characteristics of the region, we used daily climatic data sets such as the mean annual temperature and annual precipitation over the last 20 years that was developed by the Iranian Department of Meteorology. The mean annual temperature refers to the average of the maximum and minimum temperatures of a year, taking the mean average of the coldest month of the year and averaging it with the mean average of the hottest month of the year. The mean annual temperature is a valuable climatology tool that can assess the degree of weather change over the study period (Ghasemi, 2015). Annual precipitation as the sum of precipitation over each year (over the period of 2000–2019) was obtained. In addition, to characterize drought and precipitation variability, the standardized precipitation index (SPI) (Hayes et al., 1999) was used. The temporal flexibility of this index allows the assessment of drought conditions across multiple time intervals, ranging from a few weeks to a year or longer. SPI values were estimated using climatic data from the wetland synoptic station (Izeh station) for the period of 2000–2019, using Drought Indices Calculator (DrinC) version 1.7 (Tigkas et al., 2015). Positive SPI values indicate greater than median precipitation (i.e., wet conditions), and negative values indicate less than median precipitation (i.e., dry conditions).

### 2.4 Satellite data

Landsat images were favored in this study for distinguishing between land and water because there is substantial contrast between land and water in the infrared section of the electromagnetic spectrum (Guo et al., 2017). Based on the analyzed period and the availability of materials, we utilized images from the mid‐winter of 2000 to 2019 from the Landsat 7 enhanced thematic mapper (ETM) sensor (*Landsat‐7 image courtesy of the U.S. Geological Survey*) and the Landsat 8 operational land imager (OLI) (*Landsat‐8 image courtesy of the U.S. Geological Survey*). The Landsat 7 satellite has acquired images of the Earth nearly continuously since July 1999, while Landsat 8 launched in February 2013. Landsat images have a 30 m spatial and 16 days temporal resolution (Guo et al., 2017). Due to the fact that the study area is not very large, we downloaded the scenes without clouds, without using a mosaic of images from several days.

Three indices were extracted from the images (Kshetri, 2018). The normalized difference water index (NDWI) is one of the most frequently used indicators to identify open water features such as wetlands (McFeeters, 2013). This index will distinguish the water surfaces as the areas where values exceed the threshold of 0 (Xu, 2006). The normalized difference vegetation index (NDVI) is a well‐known index that has a high degree of applicability from the identification of land use/cover to monitor wetlands or the water surfaces. Both indices provide useful information about the presence/absence of water. The NDVI value varied from −1 to 1; higher NDVI values mean dense greenery and values less than 0 represent water bodies (Fan et al., 2020). The normalized difference built‐up index (NDBI) was used for the analysis of the built‐up areas. This build‐up index value lies between −1 and +1. Negative values of NDBI represent water bodies, whereas higher values represent build‐up areas (Zha et al., 2003).

### 2.5 Data analyses

The Mann–Kendall trend test was used to assess whether a set of data values is increasing over time or decreasing, and whether the trend in either direction is statistically significant. Trends in variables such as the annual precipitation, mean annual temperature, annual SPI, NDVI, NDWI, NDBI and the indices of species richness, diversity, and evenness were investigated, using the Mann–Kendall trend test (Kendall, 1975; Mann, 1945) for the period of 2000 to 2019. Based on this analysis, positive values of *Z* indicate an increasing trend, while negative *Z* values indicate decreasing trends in the time series. The value of Z is compared to the standard normal cumulative distribution to determine if there is a trend or not at the selected significance level. The critical value of *Z* from the standard normal table (at 5% significant level) is 1.96.

Generalized linear models (GLMs) implemented in R version 3.3.1 (R Development Core Team) were used to identify variables that explained variation in bird diversity. Models took indices of species abundance, richness, and diversity as the response variable, and SPI, annual precipitation and mean annual temperature, NDVI, NDBI, and NDWI were entered into the model as explanatory variables. The relative importance of explanatory variables was assessed by using hierarchical partitioning with the root mean square error as the measure of goodness‐of‐fit (Mac Nally & Walsh, 2004). Nonsignificant variables (predictors) were removed in a stepwise manner. The hier.part package version 1.0–6 was used to estimate the independent and joint contribution of each variable and identify statistically significant variables, by randomizing the data matrix 1000 times (Mac Nally, 2002). This approach addresses multicollinearity between different explanatory variables.

## 3 RESULTS

The number of wintering species inhabiting Miyangaran wetland varied among years, ranging from 10 to 48 species. The highest number (48 species) was recorded in 2007 (the year with the highest annual precipitation), and the lowest number of species was recorded in 2002. Anatidae (19 species), *Scolopacidae* (nine species), *Laridae (eight species)*, and *Charadriidae* (seven species) were the most species‐rich families within the wetland. Podicipedidae and Phoenicopteridae were the least frequent families. At the species level, the Eurasian coot (*Fulica atra*) and the black‐winged stilt (*Himantopu himantapus*) were the two most abundant species, and the greater flamingo (*Phenicopterus ruber*) was found to be the least abundant species within the wetland.

**FIGURE 2:**
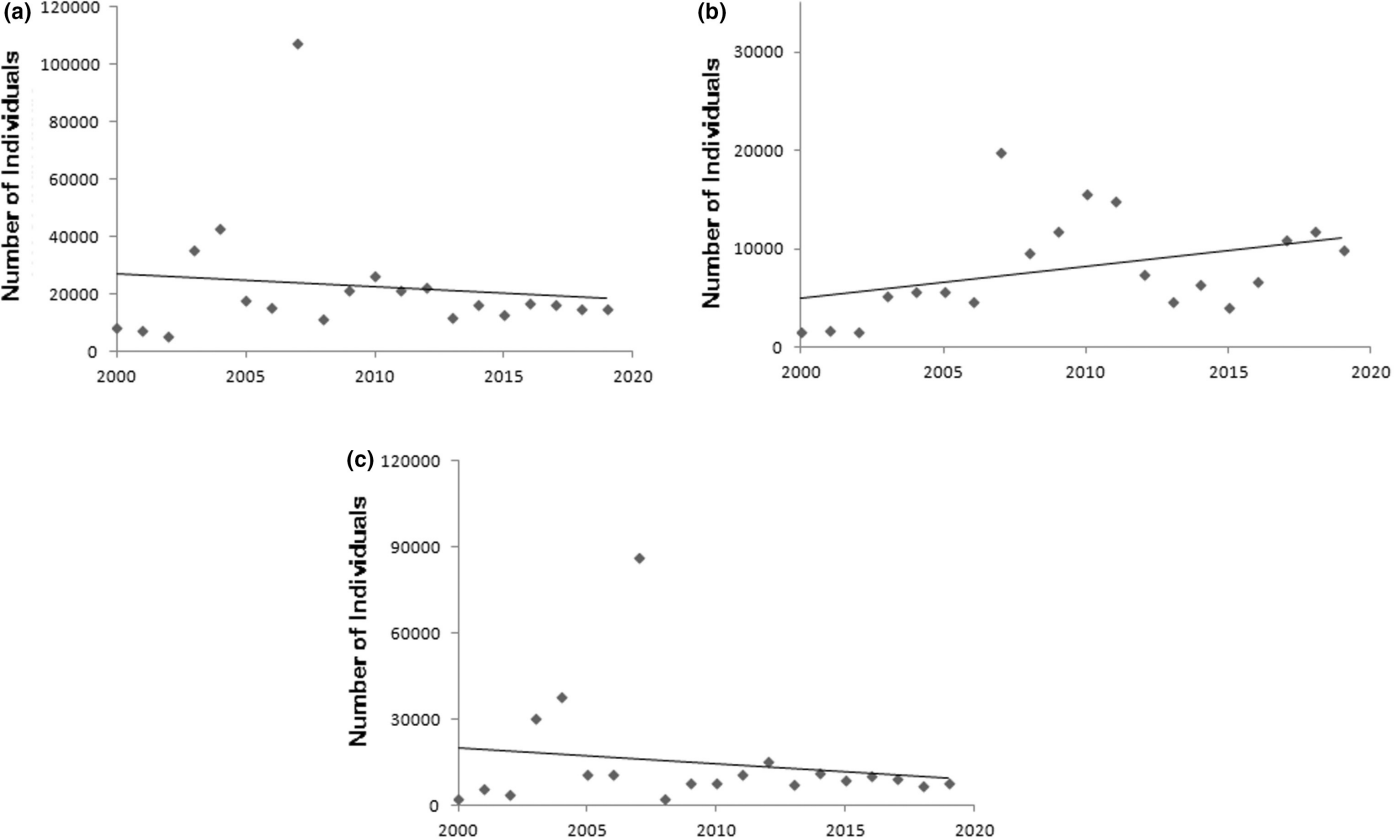
Trends in the total number of birds (a), including shorebirds (b) and waterfowl (c) recorded in Miyangaran wetland in the period of 2000 to 2019.

**FIGURE 3:**
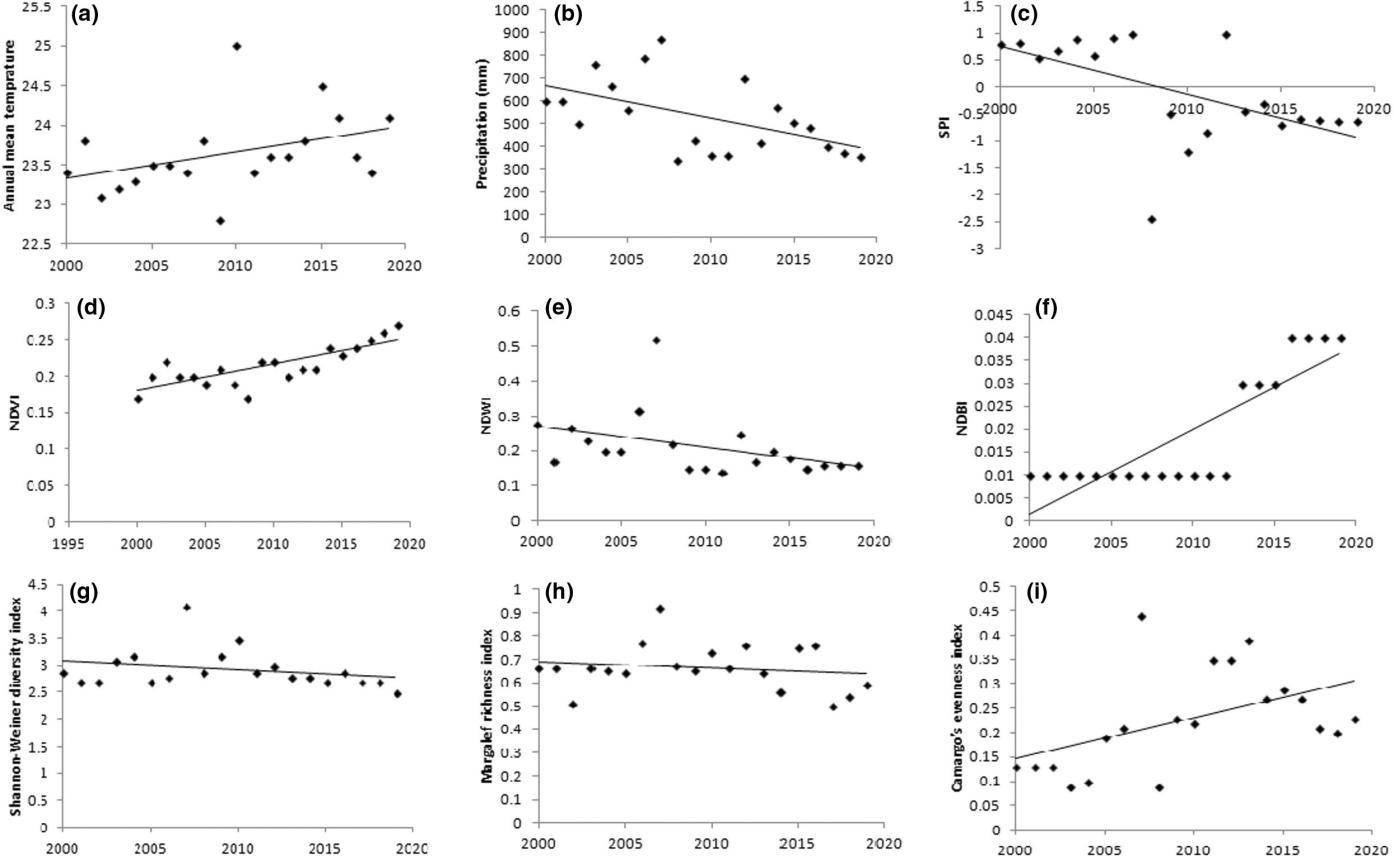
Trends of variables in the wetland over the period of 2000–2019, using the non‐parametric Mann–Kendall trend test. (a) Mean annual temperature, (b) Annual precipitation, (c) SPI, standardized precipitation index, (d) NDVI, normalized difference vegetation index, (e) NDWI, normalized difference water index, (f) NDBI, normalized difference building index, (g) Shannon‐wiener diversity index, (h) Margalef richness index, (i) Camargo's evenness index.

The total number of wintering birds (Figure 2a) showed a decreasing trend (*Z* = −1.9, *p* = .04). A significant increasing trend (*Z* = 2.6, *p* = .01) was obtained for shorebirds (Figure 2b), while waterfowl (Figure 2c) showed a decreasing trend (*Z* = −1.6, *p* = .04) over the studied period. Also, significantly decreasing trends were observed for total precipitation (*Z* = −4.2, *p* = .00), SPI (*Z* = −2.7, *p* = .02), and NDWI (*Z* = −3.7, *p* = .00) (Figure 3b,c,e). The mean annual temperature (Figure 3a) showed a slightly increasing trend over the studied period (*Z* = 1.0, *p* = .09). Significantly increasing trends in NDVI and NDBI suggest increasing agricultural and urban areas (see Section 4) around the wetland (Figure 3d,f). Despite some fluctuations, negative SPI values were observed for most of the years (Figure 3c). The Shannon–Wiener diversity index ranged from 2.5 in 2019 to the highest value of 4.1 in 2007. The highest value of the Margalef's richness index (0.92) was obtained in 2007 and the index dropped to 0.45 in 2002. Camargo's evenness index ranged from the value of 0.09 in 2002 to 0.47 in 2007 (Figure 3). The Shannon–Wiener diversity index (Figure 3g) and the Margalef's index of species richness (Figure 3h) showed nonsignificant decreasing trends (*Z* = −0.79, *p* = 0.1) over the past 20 years. The Camargo's evenness index, however, showed a significant increasing trend (*Z* = 2.46, *p* = .02) over the time period (Figure 3i).

**FIGURE 4:**
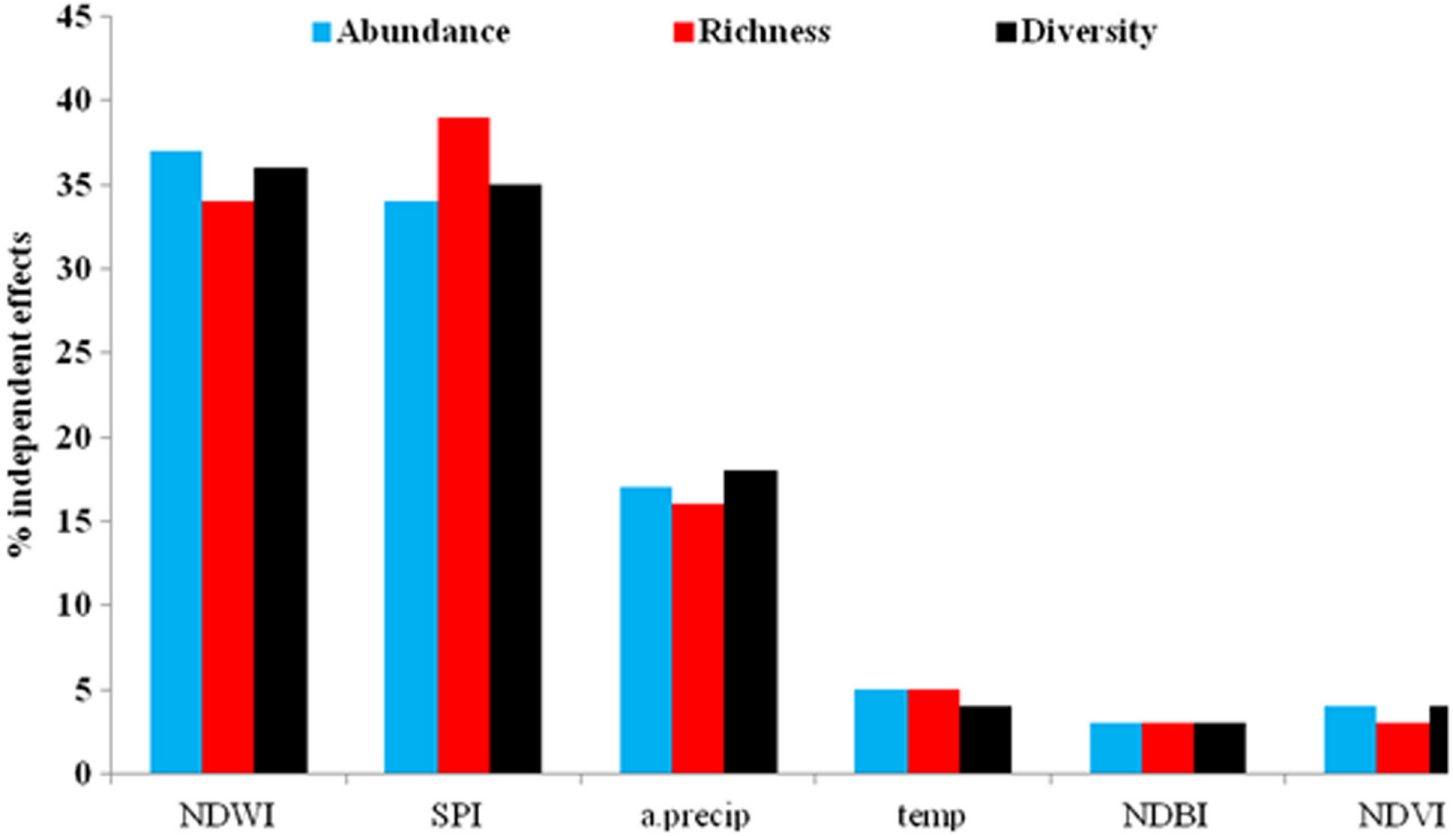
Independent contribution of the explanatory variables to bird presence in the wetland was determined by hierarchical partitioning. a. precip, annual precipitation; NDVI, normalized difference vegetation index; NDWI, normalized difference water index; NDBI, normalized difference building index; SPI, standardized precipitation index; temp, mean annual temperature.

The GLMs showed that species abundance, richness, and diversity were significantly correlated with NDWI, SPI and annual precipitation (Table 1). Hierarchical partitioning analysis also identified NDWI, SPI, and annual precipitation as the most important variables with average independent effects of 35, 36, and 17%, respectively. Mean annual temperature and NDVI with independent contributions of 5 and 3% were less important in the model (Figure 4).

**TABLE 1:** *GLM* of overall abundance and diversity in the studied wetland. Results show the explanatory variables selected for each response variable (abundance, diversity and richness) for the presence of birds.

Dependent variable	Explanatory variables	Estimate	Chi‐square	Relative contribution of variable (%)
Abundance	Intercept	0.14		
	NDWI	0.38	65.51**	42.20
	SPI	0.32	48.92**	37.10
	a.precip	0.21	8.65*	20.70
Diversity	Intercept	0.21		
	NDWI	0.31	35.84**	44.25
	SPI	0.17	19.75**	36.56
	a.precip	0.21	9.18*	19.15
Richness	Intercept	0.18		
	NDWI	0.39	55.15**	39.29
	SPI	0.29	10.58*	38.50
	a.precip	0.18	6.87*	22.12

Abbreviations: a.precip, annual precipitation; NDWI, normalized difference water index; SPI, standardized precipitation index.

Significance: **p < .01, *p < .05.

## 4 DISCUSSION

Bird data collected in the wetland showed that Miyangaran wetland supports a considerable number and diversity of wintering birds. The number and diversity of birds fluctuate between years and has significantly decreased over the past two decades. Our analyses showed that wintering birds' abundance, richness, and diversity were significantly correlated with NDWI, SPI, and annual precipitation.

Miyangaran is a closed wetland that is fed by precipitation that flows into the wetland from the surrounding mountains. Decreased rainfall and droughts in recent years have reduced freshwater inflows into the wetland. Apart from natural drought, the wetland has also suffered from the growing demand for water for agricultural activities in the Izeh plain. The enhanced use of water has reduced the habitat availability for birds. Due to the lack of accurate data, the potential effect of the surrounding agriculture on water availability was not investigated here. However, previous research showed that the number of unauthorized well drilling has increased, and the enormous water extraction has receded the overall static water level by more than 5 m (Kalantari et al., 2009). Based on our observation and data from the Department of Agriculture (Khuzestan province), the agricultural lands grew from 7150 to 9440 hectares between 1990 and 2019, and the majority of the rain‐fed lands has been converted into irrigation (Esmaeili et al., 2019).There have also been urban developments in the region, including converting 500 hectares of the wetland area into buildings and housing facilities.

In the current study, the total number of birds recorded in the wetland has decreased; however, the trends of waterfowl and shorebirds were different. An increasing trend was obtained for shorebirds, while waterfowl showed a decreasing trend over the studied period. Waterfowls are sensitive to water fluctuations, and the water level plays a major role in determining the structure of bird communities (Li et al., 2022). For example, many of large invertebrates and fish species, which are food resources for waterfowls, exist in deep waters (Xiaodan et al., 2021).The previous research also showed that some waterfowls such as sawbills and grebes have not been documented formally in the wetland since 2016, due to the decrease of water level (Behroozi‐Rad, 2017). Research showed that fluctuating or decreasing water can cause serious problems for grebe colonies, resulting in the loss of grebe nesting habitats (Ropert‐Coudert & Kato, 2009) and decreasing their population (Roesler et al., 2012).

In contrast, positive response of shorebirds might reflect increase in foraging habitats, when water levels are low. Shorebirds, mostly, prefer shallowly flooded or recently dewatered wetlands with low vegetative cover for foraging (Newcomb et al., 2014). Mudflats and associated shallowly flooded sites are ideal locations for shorebirds, increasing the number of birds by concentrating prey into a reduced water volume (Sprandel et al., 2002).

Thus, the value of wetlands as habitats is dependent on having water of sufficient quantity and quality to support wetland‐dependent birds. Conservation of wetland ecosystems requires knowledge of patterns, processes, and predicted responses to unstable events. Anticipating changes in wetland features and the potential effects on different species or taxonomic groups (Tavernia et al., 2021) are important to regulate human activities and minimize the negative impacts. Further research is needed to entirely comprehend the different mechanisms through which water availability creates different responses between groups of species.

## 5 CONCLUSION

Globally, human‐induced climate change is one of the main threats to biodiversity and its intensity is anticipated to increase in the future (IPCC, 2022). Our findings showed that changing wetland characteristics has certainly significant effects on bird communities; however, these effects may greatly differ between groups of species. Waterfowls and shorebirds with different life histories and behavioral characteristics vary in response to habitat changes.

Wetlands close to urban sites have a high chance of being converted to other types of land use. In addition, the enhanced use of water has changed the habitat availability for birds, imposing changes in the abundance, richness, and composition of bird communities. It is recommended to set a stronger emphasis on water availability through natural and artificial sources, when examining the effects of climate change on biodiversity. The link between the climatic requirements of a species and the observed population trend provides useful information for biodiversity conservation and management. Such information is needed to allow managers to understand how hydrological changes associated with climate change and human water use will affect bird assemblages.

## AUTHOR CONTRIBUTIONS

**Roghayeh Salarpour:** Conceptualization (equal); data curation (equal); methodology (equal); software (equal). **Mehrdad Ranaie:** Formal analysis (equal); methodology (equal).

## ACKNOWLEDGEMENTS

None.

## CONFLICT OF INTEREST

The authors declare that they have no competing interests.

## Data Availability

The data sets generated and analyzed during the current study are available from the corresponding author on reasonable request.
